# Low-grade developmental and epilepsy associated brain tumors: a critical update 2020

**DOI:** 10.1186/s40478-020-00904-x

**Published:** 2020-03-09

**Authors:** Rutger Juriaan Slegers, Ingmar Blumcke

**Affiliations:** 1 Faculty of Health, Medicine and Life Sciences, Maastricht University, Universiteitssingel 40, NL – 6229ER Maastricht, The Netherlands; 2 Department of Neuropathology, University Hospitals Erlangen, Schwabachanalge 6, 91054 Erlangen, Germany

**Keywords:** Pathology, Seizure, Ganglioglioma, Astrocytoma, Oligodendroglioma

## Abstract

Brain tumors represent the second most frequent etiology in patients with focal seizure onset before 18 years of age and submitted to epilepsy surgery. Hence, this category of brain tumors, herein defined as low-grade, developmental, epilepsy-associated brain tumors (LEAT) is different from those frequently encountered in adults as (A): 77% of LEAT occur in the temporal lobe; (B): the vast majority of LEAT are of low malignancy and classified as WHO I°; (C): LEAT are often composed of mixed glial and neuronal cell components and present with variable growth patterns including small cysts or nodules; (D): LEAT do not share common gene driving mutations, such as IDH1 or 1p/19q co-deletions. Characteristic entities comprise the ganglioglioma (GG), the dysembryoplastic neuroepithelial tumor (DNT), the angiocentric glioma (AG), the isomorphic diffuse glioma (IDG) and the papillary glio-neuronal tumor (PGNT), representing 73.2% of 1680 tumors collected in a large German series of 6747 patients submitted to epilepsy surgery. In the realm of exciting discoveries of genetic drivers of brain tumors new genes have been also reported for LEAT. BRAF V600E mutations were linked to GG with CD34 expression, FGFR1 mutations to DNT, MYB alterations to AG and also IDG and PRKCA fusions to PGNT, suggesting the possibility to also develop a genetically driven tumor classification scheme for LEAT. Rare availability of LEAT in a single center is a challenging obstacle, however, to systematically unravel the neurobiological nature and clinical behavior of LEAT. Other challenges in need of clarification include malignant tumor progression of LEAT entities, seizure relapse in patients following bulk tumor resection and the controversial issue of associated focal cortical dysplasia as additional pathomechanism. In order to advance our understanding and promote reliable diagnostic work-up of LEAT, we recommend, therefore, international collaboration to achieve our goals.

## Introduction

Literally every brain tumor compromising the neocortex or neuronal circuits thereof can cause a seizure and progress into chronic epilepsy [25]. This common clinical scenario was first published by Hughlings Jackson in 1882 [71]. Later the first epilepsy-associated tumor entities were described, which are nowadays seen as the most prevalent ones within the LEAT spectrum. The ganglioglioma with its biphasic composition of glial and neuronal cell elements was introduced in 1926 by Perkins, and further described by Cushing in his monograph in 1927 and by Courville in 1930 [18, 55]. The second most prevalent tumor associated with early epilepsy onset, the DNT, was first described by Daumas-Duport in 1988 in a series of 20 patients submitted to epilepsy surgery [21]. GG and DNT belong to the group of glio-neuronal tumors, as defined by the WHO. The term “long-term epilepsy associated tumors (LEATs)” was introduced by Luyken and coworker from the Bonn epilepsy center in 2003 to also recognize rare tumor entities in patients with drug-resistant long-term epilepsy that do not match with the WHO description and nosology, but are likely more close to the GG and DNT spectrum [44]. Further examples for this hitherto ongoing discovery of LEAT entities are papillary glio-neuronal tumor described in 1998 [42], angiocentric gliomas described in 2005 [73], isomorphic astrocytoma described in 2004 [6] and now referred to as isomorphic diffuse glioma [75], multinodular and vacuolating neuronal tumors of the cerebrum in 2014 [10], and polymorphous low-grade neuroepithelial tumor of the young in 2017 [34].

In a very large European multicenter cohort of almost 10,000 histopathologically defined surgical brain specimens obtained from patients submitted to epilepsy surgery [7] the group of LEAT were second most frequent to hippocampal sclerosis. A similar observation holds true for the German Neuropathology Reference Center for Epilepsy surgery (Table 1). However, the histopathological classification of LEAT remained ever challenging due to variable microscopic features including cellular components difficult to differentiate from preexisting neurons, and multiple architectural growth patterns occurring in many LEAT entities, i.e. diffuse infiltration, small cysts and/or white matter rarefaction and tumor cell nodules. Despite the large morphological variability in LEAT, common hallmarks were reported as following: A) seizure onset at a young age (< 18 years). In contrast to adult-onset seizures due to a diffusely infiltrating glioma or meningioma compromising the neocortex, seizure onset in LEAT occurs usually by the age of 13 years (Table 2). Focal neurological deficits will be, however, rare in LEAT; B) LEAT occur predominately in the temporal lobe (Table 2) of either hemisphere and have no sex preference. The neurobiological and/or neurodevelopmental basis for their preferred growth into the temporal lobe remains to be clarified; C) The histomorphological spectrum of established LEAT entities is broad (Table 2) and has significantly increased over the last three WHO classification updates [2, 3]. However, inter- and intra-rater agreement is poor for the differential diagnosis of LEAT, also affecting the WHO grading [3]. Second opinion for a histopathology review should be requested whenever a malignant cortical brain tumor, i.e. IDH1 wildtype glioma, is diagnosed in a young patient with early seizure onset as principle clinical symptom [30]; D) the vast majority of LEAT are benign and assigned to WHO I° with very few documented cases of malignant progression. However, malignant progression does occur as will be discuss below [7, 44]; E) Molecular neuropathology has revolutionized our understanding of tumor classification strategies and its impact on clinical treatment. However, these studies have very much focused on malignant tumors, such as oligodendrogliomas, glioblastomas and medulloblastomas, rather than LEAT. In addition, commonly described molecular genetic findings do not play a role in LEAT, such as IDH1R132H, 1p/19q co-deletions, TERT promotor mutations or MGMT DNA methylation. Hence, BRAF V600E, FGFR1, FGFR2, MYB/L1 and PRKCA gene alterations have been recognized in common LEAT entities and likely translate into specific subgroups. DNA methylation array analysis has supported this notion but needs further corroboration, in particular by addressing large enough and prospectively collected patient cohorts with LEAT.

**Table 1:** Brain lesion categories encountered in the German Neuropathology Reference Center for Epilepsy Surgery

Category	Numbers	Age @ Onset	Disease Duration	Age @ Surgery
HS	2144 (31.2%)	11,4	22,7	34,1
Dual	262 (3.9%)	8,6	14,6	23,3
Tumors	1680 (25.9%)	15,4	11,5	26,8
Malformations	1238 (18.3%)	6,0	12,1	18,3
No Lesion	542 (8%)	11,9	15,0	27,9
Vascular	369 (5.5%)	23,1	12,7	35,9
Scars	344 (5.1%)	9,7	14,9	25,3
Encephalitis	138 (2%)	13,2	7,7	20,7
Double	30 (0.4%)	7,0	14,8	21,4
**Total**	**6747**	**11,8**	**16,1**	**27,9**

HS-Hippocampal sclerosis; Dual-dual pathology includes HS with any other principle lesion such as tumors, malformations of cortical development (excluding associated FCD Type IIIA according to the ILAE classification of 2013); vascular malformations, glial scars (excluding postsurgical scars), or encephalitis; Tumors see Table 2; Malformations of cortical development include Focal Cortical Dysplasia (ILAE classification of 2011), polymicrogyria, hemimegalencephaly, hypothalamic hamartoma or cortical tubers; No lesion – microscopic inspection of surgical sample could not reveal any specific lesion entity, including no-HS and gliosis only; Vascular malformations include cavernoma and meningoangiomatosis in Sturge-Weber syndrome, but not ischemic stroke or hypertensive hemorrhages; glial or glio-mesodermal scars include traumatic brain injury and any pre−/peri- or postnatal stroke lesion, excluding postsurgical scaring; Encephalitis includes Rasmussen, limbic or other focal infection; Double pathology represents a combination of at least 2 principal lesions, excluding HS; Age at onset, duration of epilepsy and age at surgery in years

**Table 2:** Brain tumors encountered in the German Neuropathology Reference Center for Epilepsy surgery

Tumor entity	Number (%)	Location temporal	Age @ Onset	Disease Duration	Age @ Surgery
GG	886 (52.7)	81.6%	12,8	11,8	25,0
DNT	288 (17.1)	72.5%	14,6	10,8	25,0
PA	90 (5.4)	65%	14,9	11,9	24,2
LGNET	62 (3.7)	78.3%	17,3	12,8	29,4
IDG	40 (2.4)	42.5%	14,9	15,3	24,9
AG	13 (0.8)	53.8%	5,0	14,3	15,8
MVNT	6 (0.4)	66.7%	17,3	20,7	35,2
PGNT	4 (0.2)	75%	12,0	1,0	23,3
**Total of LEAT**	**1389 (82.7)**	**76.8%**	**13,6**	**11,8**	**25,1**
PXA	41 (2.4)	85.3%	18,6	12,2	29,7
CYSTS	34 (2)	82.4%	22,4	11,7	35,2
OLIGO	99 (5.9)	53.4%	24,2	12,4	38,1
ASTRO	70 (4.2)	57.1%	26,2	6,5	32,8
MEN	24 (1.4)	45.5%	40,7	6,2	46,1
OTHER	23 (1.4)	52.9%	17,1	9,5	26,8
**Total of non-LEAT**	**291 (17.3)**	**61.6%**	**24,2**	**10,0**	**35,1**
**Total**	**1680**	**74.0%**	**15,5**	**11,5**	**26,9**

*GG* ganglioglioma, *DNT* dysembryoplastic neuroepithelial tumor, *PA* pilocytic astrocytoma, *LGNET* low-grade neuroepithelial tumors (not otherwise specified), *IDG* isomorphic diffuse glioma, *AG* angiocentric glioma, *PGNT* papillary glio-neuronal tumor, *MVNT* multinodular and vacuolated neuronal tumor of the cerebrum, *PXA* pleomorphic xanthoastrocytoma, *CYSTS* dermoid, epidermoid or arachnoidal cysts (excluding LEAT with cystic components), *OLIGO* diffuse gliomas with oligodendroglial phenotypes, i.e. oligodendrogliomas or mixed oligoastrocytomas diagnosed before discovery of IDH1 mutations and 1p/19q co-deletions, *ASTRO* diffuse glioma with astroglial phenotypes, *MEN* meningioma, *OTHER* brain tumors of low frequency including desmoplastic infantile ganglioglioma, neurocytoma, osteoma, subependymoma, or teratoma). Location: specifying the percentage of tumors located in the temporal lobe; Age@onset: age at onset in years. Disease duration: duration of epilepsy in years. Age@surgery: age at surgery in years

### The issue of heterogeneous and not yet genetically defined LEAT entities

Our today’s body of knowledge has considerably matured into an advanced brain tumor classification scheme integrating histopathology and molecular-genetic data that can be translated into disease specific treatment regimen [43]. Unfortunately, the current WHO classification scheme of 2016 did not recommend specific molecular-genetic signatures for the neuropathological diagnosis of LEAT entities. In fact, some genetic biomarkers have been unraveled for LEAT (see below) but not yet been systematically reviewed in a large and consecutive cohort of LEAT. This dilemma further contributes to the long-lasting challenge in achieving a reliable differential diagnosis of LEAT [67] and recently confirmed by poor interobserver agreement of only 40% amongst 18 observers in a series of 25 LEAT cases using a web-based, digital microscopy platform [3]. Significant difficulties related to the differential diagnosis of ganglioglioma and DNT, when the neuronal component was difficult to differentiate from preexisting neurons overrun by glial tumor cells, either of astrocytic or clear cell/oligodendroglia-like phenotypes, or when applying the many published variants of DNT, such as simple or complex DNT [21, 69]. Notwithstanding, none of these DNT variants have been recognized by the WHO classification panel.

We concluded from these studies, that a systematic molecular-genetic approach will be mandatory to improve diagnostic reliability in LEAT and to scientifically address the many clinical challenges related to LEAT, i.e. the issue of early seizure onset, chronic epilepsy, associated focal cortical dysplasia and malignant tumor progression. Stone and coworkers confirmed that LEAT can be divided into distinct molecular subgroups using a class discovery approach [65]. One class was predominated by astrocytic differentiation patterns and BRAF V600E mutations whereas another class was enriched in FGFR1 alterations and oligodendroglial differentiation patterns. The groups were only partially concordant with histology diagnosis, however, as gangliogliomas and DNT were represented by both groups, although GG were enriched in class 1 and DNT in class 2. Similar results were found by Qaddoumi and coworkers [58] forming three molecular subgroups: a ganglioglioma-like group driven by BRAF alterations, secondly a FGFR1 group predominated by oligodendrocyte-like cells and lastly a MYB group with astrocytic and angiocentric patterns. These approaches confirmed molecular subgroups in LEAT and need corroboration in large enough cohorts to establish a reliable classification scheme for clinical diagnosis and patient stratification in future research and/or clinical trials.

#### BRAF p. Val600Glu (V600E) mutations in ganglioglioma

Davies and coworkers first described a BRAF V600E mutation in 2002 in several tumor entities, especially in malignant melanomas [22]. Later BRAF alterations were also found in low-grade (pilocytic) astrocytoma and ganglioglioma. A study from 2009 included 11 ganglioglioma and detected a BRAF V600E mutation in 3 of them [64]. This was confirmed in a larger cohort of 18 ganglioglioma of which 9 showed the BRAF V600E mutation [24]. Furthermore, the BRAF V600E mutation was screened in a cohort of 1320 nervous system tumors and detected in 18% of histopathologically diagnosed ganglioglioma (14/77), 21% of adults (11/53) and 13% of children (3/24) [62]. Notwithstanding, the BRAF V600E mutation is more common in pleomorphic xanthoastrocytoma (42/64–66%) and in 15/23 (65%) of pleomorphic xanthoastrocytoma with anaplasia, characterizing BRAF V600E as a valuable marker for gene panel diagnostics in all CNS tumors. The first BRAF V600E specific antibody was reported in 2011 (clone VE1 [15];) and is used nowadays to histopathologically screen for BRAF V600E mutations in the diagnostic work-up of formalin-fixed and paraffin-embedded tissue specimens. The VE1 antibody was scientifically explored in 71 ganglioglioma by Koelsche and coworkers in 2013 [40], detecting the mutation in 58% of these tumors (41/71). DNA sequencing in 60 of 62 cases analyzed in this study confirmed a proper detection of the BRAF mutation by VE1 antibodies. Interestingly, a BRAF V600E mutation was associated with younger patient age (compared to their previous report [62]) and not with proliferation. At the cellular level, the BRAF V600E-mutated protein was predominantly observed in neurons. In many cases mutant BRAF was also expressed by glial cells, indicating that cells carrying a BRAF mutation remain capable to differentiate into both, neuronal and glial cell lineages, and both of which represent the major cellular composition of the tumor [40]. In another study, the BRAF V600E mutation was found to be significantly associated with the expression of CD34 in 38/93 (40,8%) of ganglioglioma [56], confirming the quality of CD34 immunoreactivity as surrogate marker for GG and PXA [5, 60]. These studies of human tumor tissue were recently confirmed and further corroborated by Koh and coworkers in an animal model expressing the BRAF V600E mutation [41]. The mutation was electroporated into developing mice brain to study its cellular lineage distribution and functional consequences during tumor growth. When the mutation was successfully integrated into neuronal cell progenies 90% of the mice showed spontaneous epileptic seizures after 4 weeks postnatally, averaging five generalized, tonic-clonic seizures per day, and which could be rescued with the FDA-approved BRAF V600E inhibitor vemurafenib. The tumorigenic properties were, however, mostly due to BRAF V600E integration into the glial cell lineage. These studies experimentally confirmed our long-term proposition that tumorigenesis is related to the glial component in ganglioglioma, whereas the epileptogenic phenotype associates with post-mitotic dysplastic neurons [9].

Interestingly, and important for any future genetically driven classification scheme of LEAT, the BRAF V600E mutation was correlated with a worse recurrence-free survival in a cohort 47 GG tested, of which 18 (38%) were immunohistochemically positive [20]. In another series of 28 brainstem GG, the BRAF V600E mutation was correlated with a faster tumor regrowth compared to wild type (*p* = 0.001) and shorter progression free survival (*p* = 0.012) [16]. The notion that BRAF V600E mutations are pharmacologically addressable by next-generation kinase inhibitors, such as Vemurafenib, open an important new avenue for personalized medicine in gangliogliomas difficult to approach surgically, i.e. in the dominant hemisphere and close to eloquent cortical regions, or with histopathologically atypical or anaplastic features [27, 39, 76].

Notwithstanding, many other genetic alterations have been described in GG amongst which genetic alterations of the MAP kinase signaling pathway were most prominent. In a study of 40 GG, RAF1 (3%), KRAS (5%), NF1 (3%), FGFR1 (5%), FGFR2 (8%), ABL2 (3%), CDKN2A (8%) and PTEN (3%) were detected [54]. Up to date, these studies were driven by a histopathological stratification of included tumor tissue. The difficulties in histopathological agreement [3] and the morphological heterogeneity within many LEAT entities (see below) make this approach difficult to confirm and to use for a consensus classification. In contrast, we favor a genetically driven classification scheme of LEAT in the near future.

#### FGFR1 alterations in DNT

FGFR1 gene alterations were first reported by Jones et al. [38] in one case of pilocytic astrocytoma and simultaneously also by Zhang et al. [80] in several primary brain tumors including one DNT. A more comprehensive study revealed FGFR1 alterations in 18 of 22 DNTs studied (82%), including 9 tyrosine kinase domain duplications, eight missense single nucleotide variants and 8 FGFR1-TACC fusions. The group also noted that similar mutations were present in tumors with an oligodendroglial phenotype [58]. Rivera et al. [61] confirmed the above findings and showed 12 FGFR1 tyrosine kinase domain duplications, 10 point mutations and 3 breakpoints in a series of 25 of 43 DNTs (58,1%). Thus, FGFR1 alterations have an approximate prevalence in DNT of 58.1–82%. In a recently published DNA methylation profiling study of a small group of LEAT, there were no FGFR1 alterations found in the 5 DNT tested [3]. DNA methylation revealed, however, that the CD34-negative group of DNT were distinct from the group of CD34 positive GG, corroborating earlier findings of Stone et al. [65] and Qaddoumi and coworkers [58].

#### MYB/MYBL1 alterations in AG and IDG

**Fig. 1:**
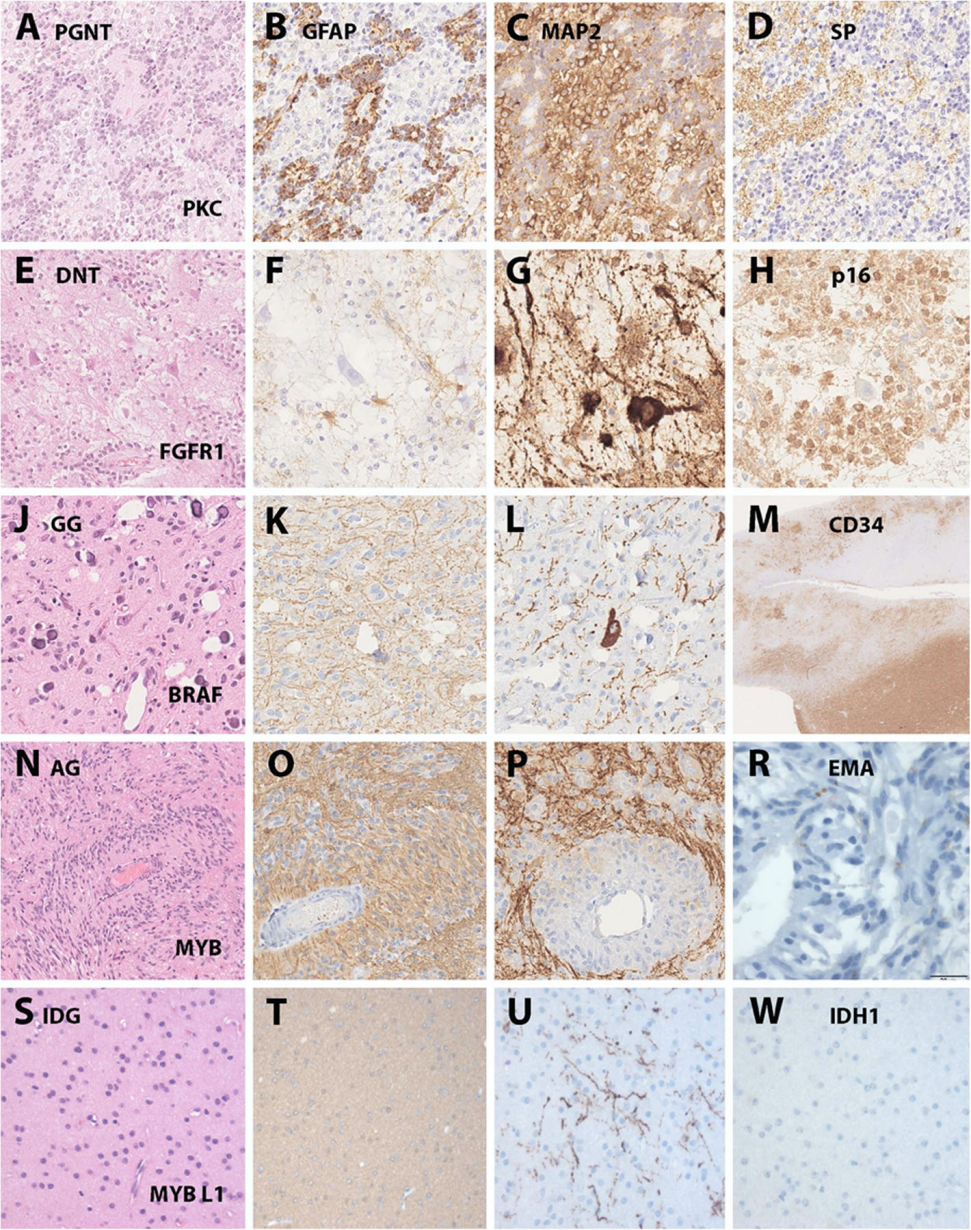
Histopathologically and genetically defined LEAT. Legend to Figure: Selected LEAT entities in which a common gene driving mutation has been discovered. **a**-**d**: a papillary glio-neuronal tumor (PGNT) with the characteristic presentation of papillary growth pattern (A – HE), glial (B – GFAP) and neuronal components (C – MAP2 and D – Synaptophysin). This tumor was included in the study by Hou et al. describing its distinct DNA methylation profile and SLC44A1-PRKCA fusion [31]. **e**-**h**: a dysembryoplastic neuroepithelial tumor (DNT) with the characteristic histological presentation of a specific glio-neuronal element (E – HE), lack of GFAP immunoreactivity in the clear-cell component (F - GFAP), floating neurons (G – MAP2) and a newly discovered p16 immunoreactivity shown in H, helpful to distinguish the DNT from other LEAT entities (unpublished observation, courtesy of Dr. Roland Coras, Erlangen, Germany). This tumor would typically present as FGFR1 altered CD34 negative DNT (not yet genetically confirmed in this tumor sample). **j**-**m**: a ganglioglioma (GG) with a characteristic glial-neuronal phenotype and small calcifications (J – HE), a predominant astroglial component (K-GFAP), dysplastic neurons (L-MAP2) and CD34 immunoreactivity in the tumor mass lesion shown in lower right corner as well as in diffusely infiltrated peritumoral grey and white matter (M-CD34). This tumor was included in the study of Blumcke et al. describing the distinct DNA methylation patterns of BRAF V600E mutated CD34 positive GG vs. CD34 negative DNT [3]. **n**-**r**: an angiocentric glioma (AG) with characteristic growth pattern around blood vessels (N-HE), a predominant astroglial phenotype (O-GFAP), enriched neuronal matrix (P-MAP2) and EMA-dots similar to ependymoma (R-EMA). This tumor showed a MYB fusion as previously described by Qaddomi et al. [58]. **s**-**w**: an isomorphic and diffusely infiltrating glioma (IDG) of low cellularity (S-HE), a predominant astroglial phenotype (T-GFAP), only few contained and pre-existing neurons (U-MAP2) and lack of IDH1R132H mutations (W-IDH mutation specific antibody). This tumor showed a MYBL1 fusion and was previously described by Wefers et al. [75]

MYB fusions have been reported as rare events in pediatric low-grade gliomas, and first described in a total of 9 tumors of which two were angiocentric gliomas [80]. This has been confirmed by Qaddomi et al. [58] who studied 15 angiocentric gliomas, and found a MYB fusion in 14/15, being predominately a MYB-QKI fusion in 13/15 patients. More recently, Wefers and coworkers studied 26 tumors histologically characterized as isomorphic astrocytoma [6] using the DNA methylation array approach. They renamed these tumors as isomorphic diffuse glioma (IDG) as they formed a separate cluster distinct from all reference cases including diffuse astrocytoma, IDH-mutant, DNT’s and ganglioglioma. The closest relation was found with a cluster of angiocentric glioma. Interestingly, 77% of IDGs also had alterations in the MYBL1- or MYB-loci, mostly representing copy number alterations or MYBL1- and MYB-fusions as shown by RNA sequencing [75]. Although IDG tumors were first reported in 2004 and not yet been recognized by the WHO panel, they likely represent a distinct (3rd) group of genetically defined LEAT and which association/familiarity with AG is in need of further clarification (Fig. 1).

#### PRKCA translocations in PGNT

The group of PRKCA altered PGNT build a 5th group of genetically defined LEAT. A PRKCA (9;17)(q31;q24) translocation was first identified in 2013 by Bridge and coworkers in two PGNT and the respective SLC44A1-PRKCA fusion detected by RT-PCR and FISH analysis [11]. This was confirmed within four pediatric cases of PGNT through FISH analysis whilst 15 PGNT mimics showed no fusion [50]. More recently, Hou et al. [31] looked at 28 PGNTs by using the DNA methylation array approach and performed a hierarchical cluster analysis comparing with 130 reference cases from 13 distinct methylation classes. 17/28 tumors clustered with classes of other established tumor entities and were probably falsely classified by a previous histopathology diagnosis. The remainder of 11 tumors formed a novel and distinct cluster, however. All of the latter group were histopathologically confirmed as PGNT, and 9/18 examined by RNA sequencing or FISH revealed a fusion with the PRKCA gene. This discovery history integrating advancing scientific methods may pave the way towards a genetically rather than histopathologically driven approach to classify LEAT in the near future.

### The issue of morphologically heterogeneous LEAT entities

Herein we will not attempt to histopathologically describe all LEAT entities. This has been tried many times by the WHO classification or neuropathology textbooks. However, it is also because the room would not be enough to recognize all variants encountered in our large clinical experience with more than 1300 LEAT samples (Table 2). As mentioned above, we expect that molecular-genetic testing will be integrated in the histopathology diagnosis of LEAT in the near future. Still for those in our neuropathology community not having access to the necessary gene panel or DNA methylation technology, we attempt to recognize and summarize common principles in LEAT cell populations and growth patterns. We will also refer to most recently described new histomorphological entities, such as the polymorphous low-grade neuroepithelial tumor of the young (PLNTY) and multinodular and vacuolating neuronal tumor (MVNT), in which the classification into molecular classes still awaits clarification.

***The neuronal component*** is per WHO definition mandatory to histologically approve a GG or DNT but can vary from almost normal and mature neurons difficult to distinguish from pre-existing neurons in the cortical ribbon, to clustered large phenotypes not anatomically or otherwise explicable, i.e. in the subarachnoidal space. Lack of specific marker proteins for these dysplastic neurons remains a challenging issue hindering better agreement amongst the neuropathological community. A number of valuable immunohistochemical marker proteins has been published to help solving this issue, see CD34, p16 or EMA in Fig. 1. We strongly recommend to apply these markers in cases where routine microscopy work-up may suggest an IDH1 wildtype glioma in a child with focal seizures and a difficult-to-classify histomorphological lesion, neither fitting into the group of hypercellular glio-neuronal neoplasia nor focal cortical dysplasia, in particular when presenting in the temporal lobe.

***The glial component*** is believed to represent the neoplastically transformed portion of the tumor, which has been elegantly confirmed in a recent molecular-biologically driven experimental animal model [41]. However, some tumors predominantly carry an astroglial phenotype and should thus classify rather into the group of ganglioglioma, isomorphic or pilocytic glioma. When an oligodendroglia-like/clear-cell phenotype prevail, a diagnosis of DNT being conform with the WHO classification should refer to a nodular tumor with a specific glio-neuronal element [66]. It has to be emphasized, however, that clear-cell morphologies can also occur in GG and PLNTY, in particular those characterized by CD34 immunoreactivity. Astrocytic phenotypes with an angiocentric presentation or ependymal-like architectures should rather classify into the group of AG (Fig. 1).

***Growth patterns*** largely vary in most LEAT entities including small cysts, white matter rarefaction, diffuse infiltration patterns or cell clusters remote from the tumor mass, and nodular growth. In contrast to many textbook edits and MRI interpretations, LEAT can show diffuse infiltration. However, proliferation indices remain usually very low in a given surgical sample. It is hypothesized, therefore, although not yet scientifically confirmed, that the early neurodevelopmental nature of LEAT will allow tumor satellite clusters to dissolve into cortical areas remote from the primary mass lesion. We also believe that this infiltration pattern has the ability to promote seizure onset remote from the MRI visible tumor lesion, but this also needs scientific confirmation.

All of the above-mentioned principles may expose themselves in even yet unprecedented and unpublished phenotypes, contributing to the growing list of published LEAT entities. We believe and hypothesize herein, that the early “developmental” origin of the tumor, most likely in prenatal periods, as well as continuous electric bombardment of postnatally developing and maturing neocortex contribute foremost to the broad histomorphological spectrum of LEAT rather than representing distinct and clinically meaningful entities, i.e. with a higher risk for seizure relapse or malignant tumor progression. Our better understanding of genetic etiologies and epigenetic modifications thereof will eventually prove or disprove this hypothesis in times to come.

#### New histomorphological entities

Polymorphous low-grade neuroepithelial tumor of the young (PLNTY) is an entity first described by Huse et al. in 2017 [34]. The group presented ten patients with a young age at diagnosis (between 4 and 32 years old) and tumors with infiltrative growth patterns, a predominant oligodendoglioma-like glial cell component and intense CD34 immunoreactivity as most common features. Three out of eight tested tumors showed a BRAF V600E mutation, one showed a FGFR3 fusion and three had a FGFR2 fusion. Such FGFR2 fusions have not yet been detected in any other LEAT category and might qualify as a distinctive feature in the near future. The mixed molecular-genetic background, however, including BRAF and FGFR genes described above, makes PLNTY yet difficult to stratify into a classification scheme led predominately by gene driving mutations rather than histomorphological features. However, DNA methylation profiling showed similarities with the methylation class of ganglioglioma but also suggested that PLNTY may form a separate group [34]. Further studies will help to clarify this issue.

In 2013 Huse et al. described multinodular and vacuolating neuronal tumors (MVNT) in ten patients [33], which was confirmed 2014 by Bodi et al. in 2 additional patients [10]. MVNT is defined nowadays by the WHO as a benign tumor associated with seizures. These tumors have a typical radiological pattern described as FLAIR and T2-WI hyperintense lesions, clustered in multiple small nodules, affecting subcortial white matter surrounded by normal-appearing parenchyma [13, 28]. The WHO has subsumed this variant, therefore, into the group of gangliocytoma and not yet assigned a grading [43]. In a series of seven MVNTs no BRAF V600E mutations were found but one case showed a FGFR2 fusion [17]. In another series of eight MVNTs genetic alterations were found in BRAF other than V600E, MAP2K1 and FGFR2 (2/8, 5/8 and 1/8, respectively) [53]. As mentioned above for PLNTY, the diverse molecular landscape of findings reported in the literature make it difficult to align this entity into a genetically driven classification scheme and more studies are needed to clarify the etiology of this hitherto histomorphologically defined entity. Lack of cell proliferation and lack of expansive or infiltrative growth reinforced also the debate whether MVNT should align with malformations of cortical development or cortical dysplasia rather than with a neoplasm [68].

### The issue of brain-tumor related epilepsy and associated focal cortical dysplasia

Whereas our knowledge of molecular pathways driving neoplastic cell growth and malignant progression has substantially matured, the issue of ictogenesis, i.e. why and how a seizure occurs in a patient with a brain tumor, and epileptogenesis, i.e. turning a normal into an epileptic brain prone to unprovoked recurrent seizures, still awaits clarification. Brain tumors cause about 10 to 15% of all adult-onset and 0,2 to 6% of all child-onset epilepsies [7, 12, 70]. Notwithstanding, many alterations have been reported in human peritumoral brain tissue which have the potential to dramatically alter neuronal and glial homeostasis and the microenvironment in favor of a pro-epileptogenic state [19, 51, 70]. These studies also proposed candidate therapeutic regimen for treatment of patients with brain-tumor related epilepsy [32]. Two main hypotheses have been proposed; the tumorocentric and the epileptocentric approach [52]. The tumorocentric approach states that the epileptic activity derives from the tumor itself which was recently confirmed by experimental work of Koh et al. in neurons transfected with the BRAF V600E mutation in vivo [41]. The epileptocentric approach provides evidence that the infiltrated peritumoral neocortex is key for tumor-related epileptic activity, due to glioma-related glutamatergic and γ-aminobutyric acid changes leading to epileptogenicity [52]. The neurotransmitter glutamate is used by glioma cells as a “tumor growth factor”. Altered expression of glutamate transporters by the tumor cells, including the cystine-glutamate transporter (xCT) system, also increases concentrations of extracellular glutamate contributing to epileptic discharges, tumor proliferation and neurotoxicity [32]. However, such experimental studies have been performed so far only on histopathologically diagnosed low-grade glioma and not LEAT.

These considerations lead to the other important issue which is how to achieve complete postsurgical seizure control. Many studies have confirmed that active and medically uncontrolled epilepsy significantly increases the risk of sudden death [23]. However, LEAT were amongst the best candidates for complete postsurgical seizure control [7]. Planning for epilepsy surgery needs to take into consideration, therefore, not only any MRI visible lesion but to also resect the ictal onset zone. Scalp EEG may be misleading when the ictal onset is buried deep in the cortex, i.e. in temporo-mesial structures, and intracerebral procedures were increasingly used to delineate the epileptogenic as well as ictal onset zones. Correlations with histopathology have not been performed, however, in a systematic way to allow any conclusion about the structural and/or molecular correlate. One result of this dilemma is the ongoing discussion about Focal Cortical Dysplasia associated with LEAT, hitherto classified as FCD IIIB by the ILAE classification scheme from 2011 [8]. Histopathology patterns of such FCD have never been scientifically defined. As a consequence, scientific reporting of the prevalence of dysplastic neocortex around LEAT, including ganglioglioma and DNT, varies to a great extent by 25 to 75% of the cases [1, 4, 26, 37, 48, 57]. The authors expect that ongoing molecular-genetic studies will help to clarify if these cases represent true FCD or pro-epileptogenic molecular interactions of the tumor with surrounding peritumoral brain tissue.

### The issue of malignant progression in LEAT

The proposal for surgical treatment in a young patient presenting with an MRI stable or only slowly growing LEAT, that is initially well controlled by antiepileptic drugs, may raise considerable concern in patient management. We strongly advocate, however, to counsel for complete neurosurgical resection and a histopathological diagnosis to confirm the benign nature of a neoplasia. As a matter of fact, malignant progression has been reported in LEAT [46], although at very low frequency and we do not have approved biomarker or molecular signatures predictive for such malignant progression, yet. Reported prevalence of malignant progression differ among the LEAT entities. The most common tumor within this group, the ganglioglioma, has an estimated chance of 3% for malignant progression whilst the second most common tumor entity, the DNT (Table 2) has near to 0 % chance for dedifferentiation [36, 45, 49, 77]. This highlights the importance of a reliable differential diagnosis as it will directly influence patient management and also therapeutic regimens.

#### Anaplastic ganglioglioma

Reported cases of LEAT with confirmed malignant progression were mostly addressing ganglioglioma. In a retrospective analysis of 55 pediatric ganglioglioma cases in a single center setting 53 had a ganglioglioma and 2 an anaplastic ganglioglioma at time of diagnosis. After a mean follow-up time of 9,5 years 25 showed tumor progression and 6 transformation to a higher grade after which a median survival of 9.1 months was reported [78]. Zanello et al. [79] analyzed 18 anaplastic ganglioglioma in a retrospective series forming 8% of their ganglioglioma cohort of 222 patients. They also looked at molecular alterations and found BRAF V600E in 39%, hTERT promotor mutations in 61%, p53 accumulation in 39%, ATRX loss in 17% and p.K27M H3F3A mutations in 17% of the cohort. A median progression-free survival of 10 months and a median overall survival of 27 months was reported within this cohort [79]. We suggest, however, re-examine these tumor samples with a more objective measure, such as DNA methylation, as they may have been histopathologically assigned falsely into the group of GG (see the many pitfalls listed above) and belong rather into the group of malignant glioma.

#### Malignant dysembryoplastic neuroepithelial tumors

DNTs are considered as truly benign with a near to zero chance of malignant progression. In our own experience with more than 280 DNT, all of which can be classified as classic variants with nodular growth, a specific glio-neuronal element and no tumor-cell related CD34-immunoreactivity (Table 2), we have not observed a single patient with tumor relapse due to malignant progression. Heiland and coworkers [29] presented a case, however, which was characterized as DNT and which relapsed 5 years after tumor resection as a glioblastoma (GBM). DNA methylation analysis of the tissue samples showed a methylation pattern distinct from typical GBM. Malignant progression of DNTs were reported earlier by Ray and coworkers in 2009 [59] or Thom and coworkers in 2011 [69]. Notwithstanding, these reports were published before the era of DNA methylation classifier [14], which leaves us with the notion that malignant transformation can occur in every tumor but need particular attention, re-review if necessary, and current state-of-the-art molecular-genetic re-assessment when occurring in a DNT.

#### Pleomorphic xanthoastroctyoma (PXA)

PXA are semi-malignant brain tumors sharing molecular and morphological commonalities with LEAT. We have not addressed PXA as bona fide LEAT entity, however, due to their semi-benign nature with WHO tumor grading of atypic II° and anaplastic III° subtypes. In addition, disease onset had a mean age of 18,6 years in 41 PXA collected at the German Neuropathology Reference Center for Epilepsy Surgery (see Table 2). Histomorphological similarities between PXA and LEAT include, however, the broad spectrum of different tumor cell components and growth patterns as described above. Reifenberger and coworkers reported an intense CD34 immunoreactivity in 73% of their PXA, which was more common in grade II (84%) than in grade III tumors (44%) [60]. Another similarity to GG is the BRAF V600E hotspot mutation identified in 71.4% of 167 published PXA [47], more often in grade II than in grade III tumors (75% and 47,4%, respectively [35, 63]. A homozygous deletion of CDKN2A/B, corresponding to loss of 9q21.3, is a rather distinctive molecular feature of PXA [74]. In a study of 24 PXA and 14 anaplastic PXA, CDKN2A/B deletions were identified in 83 and 93%, respectively [72]. Knowledge of and access to the molecular-genetic landscape of these brain tumors will be most helpful, therefore, to classify tumor entities into similar molecular classes despite their variable histomorphological phenotypes. It awaits further studies, however, to precisely define also clinically meaningful entities.

## Conclusion

The histopathological spectrum of LEAT is heterogeneous, both morphologically and genetically! Even with currently available molecular genetic markers such as BRAF V600E and FGFR1 and immunohistochemical surrogate markers, such as CD34 and p16, there is still a poor inter-rater agreement in the histopathological diagnosis. Any effort should be taken, therefore, to improve and standardize our criteria and terminology, and to extent the use of molecular genetic diagnostic tools over a histomorphology-based classification to specify clinically meaningful tumor entities within the LEAT spectrum. There is also a small risk for malignant transformation of LEAT, but currently not predictable with any available biomarker. A large enough and unselected, consecutive cohort of LEAT is a mandatory pre-requisite for our further progress in the field and will require international collaboration efforts.

## Data Availability

The datasets generated during and/or analyzed during the current study will be made available from the corresponding author on reasonable request.

## Abbreviations

AG: Angiocentric glioma WHO I°
ATRX: Alpha-thalassemia/mental-retardation syndrome-X-linked
BRAF: B-Raf Proto-oncogene, serine/threonine kinase
CNS: Central nervous system
CNV: Copy number variants
DNT: Dysembryoplastic neuroepithelial tumor WHO I°
EEG: Electroencephalography
FCD: Focal cortical dysplasia
FGFR1: Fibroblast growth factor receptor 1
GBM: Glioblastoma multiforme WHO IV°
GG: Ganglioglioma WHO I°
HS: Hippocampal sclerosis
IDG: Isomorphic diffuse glioma
K27M H3F3A: Lysine 27 replacement with methionine in histone H3 variant H3.3
LEAT: Low-grade, developmental and epilepsy-associated tumor
LGNET: Low-grade neuroepithelial tumor (NOS)
MGMT: Methylguanine methyltransferase
MRI: Magnetic resonance imaging
MVNT: Multinodular and vacuolated neuronal tumor of the cerebrum
MYB/MYBL1: MYB proto-oncogene (like 1), transcription factor
PA: Pilocytic astrocytoma WHO I°
PGNT: Papillary glio-neuronal tumor WHO I°
PRKCA: Protein kinase C alpha
PXA: Pleomorphic xanthoastrocytoma WHO II°
TERT: Telomerase reverse transciptase
xCT: Cystine-glutamate transporter
